# Surgical and Transcatheter Approach of a Failed Mitral Valve Repair: A Comprehensive Review on Selecting the Most Suitable Approach

**DOI:** 10.3390/jcm15124847

**Published:** 2026-06-22

**Authors:** Roberto Nerla, Martina Mandas, Gianluca Pillitteri, Elisa Mikus, Niki Bernardoni, Angelo Squeri, Davide Pacini, Carlo Savini, Fausto Castriota

**Affiliations:** 1 Interventional Cardiology Unit, Maria Cecilia Hospital GVM Care and Research, 48033 Cotignola, Italyfcastriota@msn.com (F.C.); 2 Department of Experimental Diagnostic and Surgical Medicine (DIMEC), University of Bologna, 40126 Bologna, Italy; 3 Cardiac Surgery Unit, Maria Cecilia Hospital GVM Care and Research, 48033 Cotignola, Italy; 4 Cardiac Surgery Unit, Policlinico Sant’Orsola, University of Bologna, 40138 Bologna, Italy

**Keywords:** mitral valve repair, mitral regurgitation, surgical mitral valve re-repair, mitral valve replacement, transcatheter edge-to-edge repair, transcatheter mitral valve replacement, valve-in-valve, valve-in-ring, heart team

## Abstract

Mitral valve regurgitation is the second most common valvular heart disease in Europe, and an estimated 10% of individuals older than 75 years have severe mitral regurgitation. Mitral valve repair is the preferred strategy to treat mitral regurgitation and is associated with better outcomes than mitral valve replacement. Despite the proven efficacy of surgical repair, available data in functional aetiologies reported a non-negligible rate of echocardiographically detected severe mitral regurgitation within ten years of the index procedure, in some cases resulting in redo interventions. Data on the optimal management of patients with failed mitral repair remain limited. The aim of this review is to present the available approaches for treating failed mitral valve repair and to describe criteria for selecting the most appropriate strategy on the basis of the underlying mechanism of repair failure, with respect to possible surgical re-repair and novel transcatheter edge-to-edge repair techniques in the presence of favourable mitral valve anatomies.

## 1. Introduction

Mitral valve regurgitation (MR) is the second most common valvular heart disease in Europe, and an estimated 10% of individuals older than 75 years have severe MR [1]. According to the predominant mechanism, MR is classified as primary or secondary. Primary MR is characterized by leaflet pathology, most frequently driven by myxomatous degeneration of the mitral valve apparatus, whereas secondary or functional MR (SMR) develops as a consequence of left ventricular and mitral apparatus remodeling. Mitral valve repair (MVr) is the preferred strategy to treat MR and is associated with better outcomes than mitral valve replacement (MVR) [2]. The primary objective of MVr is to restore annular geometry, optimize leaflet motion, and ensure durable leaflet coaptation [3]. Despite the proven efficacy of surgical repair, available data in functional etiologies report nearly 30% recurrence of echocardiographic evidence of severe MR within ten years of the index procedure [4,5], resulting in a non-negligible number of redo interventions, with reoperations now accounting for more than 10% of all mitral valve surgeries [5].

**Figure 1:**
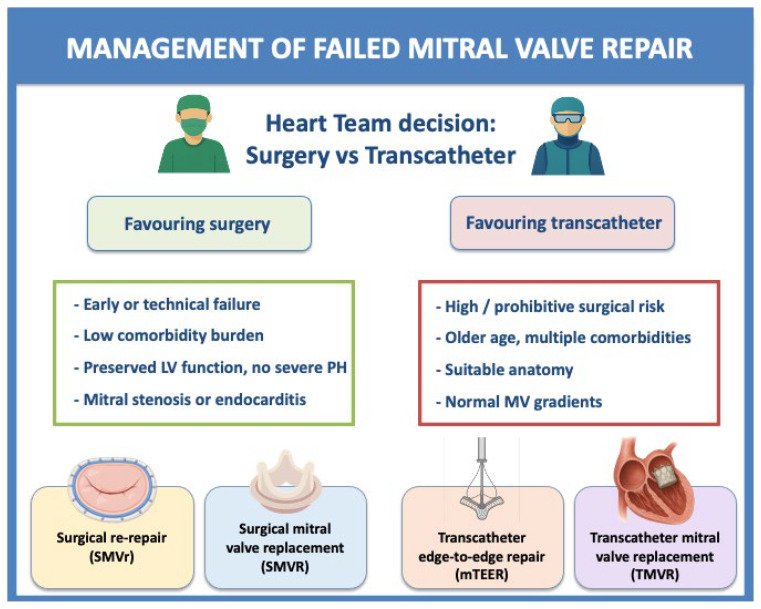
Surgical and transcatheter options to treat failure of mitral valve repair: clinical and anatomical features for a tailored approach. LV = left ventricle; mTEER = mitral transcathter edge-to-edge repair; MV = mitral valve; PH = pulmonary hypertension; SMVr = surgical re-repair; SMVR = surgical mitral valve replacement; TMVR = transcatheter mitral valve replacement.

Reoperation for failed repair is associated with higher risk, and recurrent (≥moderate) MR after surgical repair is associated with substantial mortality and morbidity [6,7]. However, data on the optimal management of patients with failed MVr remain limited. The aim of this review is to present the available approaches for treating failed MVr and to describe criteria for selecting the most appropriate strategy on the basis of the underlying mechanism of repair failure. Since no randomized controlled trials directly compare SMVr, SMVR and percutaneous strategies, available evidence is predominantly observational and therefore inherently affected by selection bias, operator preference, and referral patterns, with transcatheter therapies being more common in frail patients. However, analyzing anatomical contraindications and results of either approach might be helpful to define a tailored treatment for each patient (Figure 1).

Mitral valve repair failure is defined as the recurrence of moderate or greater MR, mitral stenosis, hemolysis, or infective endocarditis, regardless of symptoms, after MVr [8].

There are two main classifications of repair failure, based on the timing of presentation and on the mechanism of failure. According to timing, repair failure is classified as *early* if it occurs within one year after surgery and *late* if it occurs beyond one year.

*Early failure* is frequently related to suture or annuloplasty dehiscence but can also result from systolic anterior motion (SAM) of the mitral valve or early adverse left ventricular remodeling. These failures are often characterized by correctable technical issues, allowing re-repair in approximately 40% of cases [9].

*Late failure* is more commonly caused by progression of mitral valve disease and reactive changes following the initial repair, such as fibrosis or leaflet scarring. Patients presenting with late failure are more frequently treated with valve replacement [9]. 

Repair failure can also be classified according to the predominant mechanism as technical or valve-related, as shown in Table 1. Valve-related failure tends to occur later [10] and more often requires valve replacement [11,12].

The primary aim of this review is to provide a comprehensive overview of the currently available surgical and transcatheter treatment strategies for patients presenting with failed mitral valve repair. The secondary aims included the following: (1) to analyze the mechanisms and timing of mitral valve repair failure and their implications for treatment selection; (2) to summarize the available evidence regarding outcomes of surgical mitral valve re-repair, surgical mitral valve replacement, transcathtere edge-to-edge repair and transcatheter mitral valve replacement; (3) to identify clinical and anatomical features that may guide a tailored decision-making process in selecting the best therapeutical option.

## 2. Methods

This review was conducted as a structured, quasi-systematic review of the available literature on the management of failed mitral valve repair. The methodology was designed to ensure a comprehensive and reproducible identification of relevant studies while allowing flexibility in the qualitative synthesis of heterogeneous data. A systematic search of the electronic databases PubMed/MEDLINE, Embase, and the Cochrane Library was performed to identify studies published up to January 2026. The search strategy combined Medical Subject Headings (MeSH) and free-text terms, including: “mitral valve repair failure”, “recurrent mitral regurgitation”, “redo mitral surgery”, “mitral valve re-repair”, “mitral valve replacement”, “transcatheter edge-to-edge repair”, “MitraClip”, “transcatheter mitral valve replacement”, “valve-in-ring”, and “valve-in-valve”. Reference lists of selected articles and relevant reviews were also manually screened to identify additional studies.

Studies were considered eligible if they reported clinical, procedural, or imaging outcomes of surgical (SMVr, SMVR) or transcatheter (mTEER, TMVR) interventions in patients with failed mitral valve repair. Observational studies, registries, and meta-analyses were included, whereas case reports and small case series (<10 patients) were selectively included only when providing relevant technical or anatomical insights. Study selection was performed by the authors based on title and abstract screening, followed by full-text evaluation. Data extraction focused on study design, patient characteristics, mechanism of repair failure, type of intervention, procedural success definitions, and clinical outcomes. Given the absence of randomized controlled trials in this field, the available evidence consists predominantly of observational data, which were critically appraised with particular attention to selection bias, referral bias, and variability in endpoint definitions across studies.

## 3. Surgical Mitral Valve Re-Repair (SMVr)

Surgical mitral valve re-repair (SMVr) is safe, durable, and may confer a survival benefit over valve replacement, particularly when failure occurs within one year of the index surgery. When feasible, SMVr should therefore be the preferred strategy. In contemporary series, it is associated with a lower in-hospital mortality (9.8%) than MVR with a bioprosthesis (12.7%) or a mechanical prosthesis (12.2%) [13] and with significantly improved long-term survival compared with MVR, with outcomes approaching those of first-time mitral valve surgery [14]. When evaluating patients for mitral valve re-intervention, favorable and unfavorable factors for re-repair should be considered, including the time interval between the index operation and re-intervention, patient age, comorbidities, and operative findings, as summarized in Table 2. The mechanism of failure can also help in estimating the likelihood of successful re-repair [11,15,16,17]. A shorter time to reoperation is a major factor favoring re-repair: in one series, the median time to reoperation was 1.3 years among re-repairs versus 3.9 years among replacements [9]. Operative findings such as suture dehiscence and adverse ventricular remodeling, which are more common in early failures, are strongly predictive of effective re-repair. Patients selected for valve re-repair are generally younger, have fewer cardiac and noncardiac comorbidities, and less frequently present with advanced New York Heart Association (NYHA) functional class symptoms than those undergoing replacement. They are also less likely to require concomitant procedures, particularly coronary revascularization, tricuspid repair, or atrial fibrillation surgery [9] (Table 3).

Thus, the feasibility of re-repair is significantly influenced by patient age and comorbidities, the etiology of recurrent MR, the presence of complex disease, and the timing of reoperation. Conversely, the presence of mitral stenosis or endocarditis is strongly associated with the need for valve replacement.

Importantly, mechanisms of repair failure and the likelihood of successful re-repair differ substantially between *degenerative* and *functional* mitral valve disease. In degenerative MR, recurrent regurgitation is more frequently related to leaflet or chordal pathology, including progression of myxomatous degeneration, new prolapse segments, or chordal rupture, conditions that are often technically amenable to repeat repair. Conversely, in functional MR, recurrent regurgitation is more commonly driven by progressive left ventricular remodeling, papillary muscle displacement, leaflet tethering, and annular dilatation, resulting in a more complex and dynamic substrate with lower repair durability. Accordingly, patients with functional MR more frequently require valve replacement or transcatheter therapies, particularly in the presence of advanced ventricular remodeling and unfavorable tethering geometry. It could be said that the key determinant of repair failure and of the feasibility of surgical re-repair is LV remodeling. Changes in LV geometry following the index procedure may significantly alter the spatial relationship between papillary muscles and mitral leaflets, leading to functional leaflet malcoaptation even in the absence of primary leaflet pathology.

In particular, adverse LV remodeling may result in displacement of the papillary muscles and increased tethering forces, thereby reducing leaflet coaptation and contributing to recurrent mitral regurgitation. In this context, the phenomenon of “pseudo-elongation” of artificial chordae has been described as a relevant mechanism of early or mid-term failure [18]. As the left ventricle undergoes reverse remodeling after successful repair, the relative length of previously implanted neochordae may become excessive, thus resulting in recurrent leaflet prolapse. Conversely, progressive LV dilatation may exacerbate leaflet tethering and further impair coaptation. These dynamic interactions between ventricular geometry and subvalvular apparatus highlight the importance of ventricular remodeling in determining repair durability.

From a surgical perspective, recognition of LV remodeling and pseudo-elongation is crucial, as these mechanisms are often amenable to re-repair through targeted techniques such as chordal shortening, neochordal reimplantation, or adjustment of leaflet coaptation geometry. Accordingly, these conditions are generally associated with a higher likelihood of successful re-repair compared with advanced degenerative or fibrotic valve disease [19].

The most frequently used approaches include leaflet resection, posterior leaflet sliding, and neochordal implantation, as summarized in Table 4.

A distinct and particularly challenging subgroup is represented by patients presenting with clinically significant hemolysis following failed mitral valve repair. Although relatively uncommon, hemolysis is typically associated with high-velocity, eccentric regurgitant jets caused by partial annuloplasty dehiscence, leaflet perforation, or residual/recurrent prolapse, leading to mechanical destruction of red blood cells through shear stress mechanisms [20,21]. From a clinical standpoint, these patients often present with hemolytic anemia requiring transfusions, elevated lactate dehydrogenase levels, reduced haptoglobin, and, in some cases, progressive renal dysfunction, which may substantially increase overall procedural risk. [20]. Therapeutic decision-making in this setting is particularly complex. Surgical correction—either re-repair or replacement—has traditionally been considered the most definitive strategy, as it directly addresses the underlying mechanical substrate of hemolysis and is associated with prompt resolution in the majority of cases [21]. However, redo surgery may carry significant morbidity, especially in patients with multiple prior interventions or advanced comorbidities. The role of transcatheter therapies in this subgroup remains less clearly defined. mTEER may be effective when hemolysis is driven by a single, well-localized regurgitant jet amenable to leaflet approximation; however, its efficacy is less predictable in the presence of multiple jets, complex flow patterns, or significant ring dehiscence. Importantly, partial reduction of MR may be insufficient to resolve hemolysis, even when echocardiographic results are deemed satisfactory.

## 4. Surgical Mitral Valve Replacement in Failed Repair (SMVR)

Surgical mitral valve replacement (SMVR) remains the only universally applicable option for recurrent MR not amenable to re-repair. Although SMVR after previous repair is associated with poorer outcomes than primary surgery, it is necessary in patients requiring reoperation for mitral stenosis, with or without regurgitation, bileaflet prolapse, severe degenerative progression of native disease, endocarditis (which contributes substantially to the higher mortality associated with re-replacement) [22], multivalve procedures, or unfavorable anatomy for re-repair (Table 3). Re-replacement forces patients and physicians to choose between two imperfect options: mechanical valves, which require lifelong anticoagulation, and bioprosthetic valves, which carry a risk of structural valve deterioration and negatively influence left ventricle remodeling. Unlike mitral valve repair, valve replacement may disrupt the physiological continuity between the mitral annulus, leaflets, chordae tendineae, and papillary muscles, particularly when the subvalvular apparatus is not preserved. This loss of annulo–ventricular continuity has been associated with impaired LV systolic function and adverse ventricular remodeling, ultimately affecting long-term outcomes [22]. Furthermore, prosthetic valves—especially bioprostheses—introduce non-physiological hemodynamic conditions, including altered transmitral flow patterns and reduced effective orifice area compared with native or repaired valves. These factors may contribute to increased LV filling pressures and progressive ventricular remodeling over time. In the mitral position, such effects may be more pronounced due to the higher hemodynamic load and the sensitivity of the left ventricle to changes in preload and afterload conditions [23]. Consequently, degeneration of biological prostheses in the mitral position progresses more rapidly than in the aortic position [23].

The choice between mechanical and tissue prostheses in redo surgery is based on a combination of factors, including age, eligibility for anticoagulation, comorbidities, and desire for pregnancy. Several independent predictors of mortality during redo MVR have been identified, including moderate to severe renal dysfunction, prior stroke or transient ischemic attack (TIA), left ventricular systolic dysfunction with a left ventricular ejection fraction (LVEF) < 40%, urgent or emergent procedures, and lack of subvalvular apparatus preservation at the index surgery [22]. The incidence of complications after redo replacement, including supraventricular arrhythmias, sepsis, acute renal failure requiring renal replacement therapy, and stroke, is significant [24,25]. An operative mortality of approximately 8% has been reported, together with increased blood transfusion requirements and longer mechanical ventilation compared with the redo MVr cohort, in a study including 305 patients who underwent reoperation following a previous repair [26].

## 5. Percutaneous Approach: Transcatheter Mitral Edge-to-Edge Repair (mTEER)

**Figure 2:**
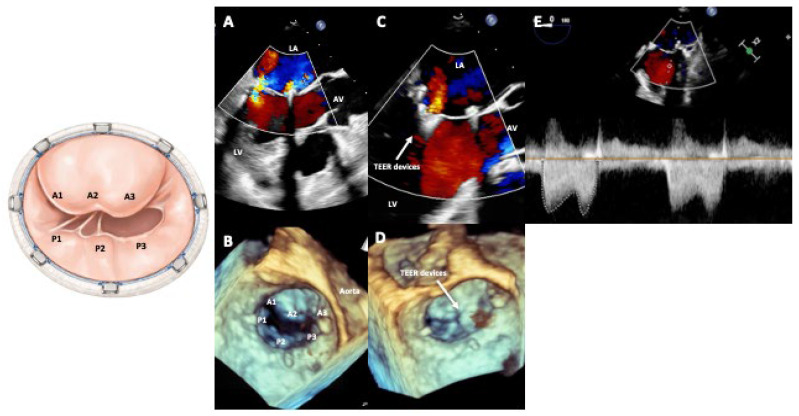
Transcatheter Edge-to-Edge Repair in Annuloplasty Failure. (**A**) Color Doppler TEE showing severe functional mitral regurgitation with a double regurgitant jet directed into the left atrium (LA). (**B**) Three-dimensional TEE en face left atrial view of the mitral valve before intervention with impaired coaptation involving the central scallops (A2–P2) and adjacent segments. (**C**) Post–transcatheter edge-to-edge repair (TEER) color Doppler image showing marked reduction of mitral regurgitation after device implantation (arrow). (**D**) Post-procedural three-dimensional en face view from the left atrium illustrating successful leaflet approximation with TEER devices (arrow). (**E**) Continuous-wave Doppler across the mitral valve after TEER demonstrating preserved transmitral flow with a low mean gradient. LA = left atrium; LV = left ventricle; AV = aortic valve; TEER = transcatheter edge-to-edge repair.

**Figure 3:**
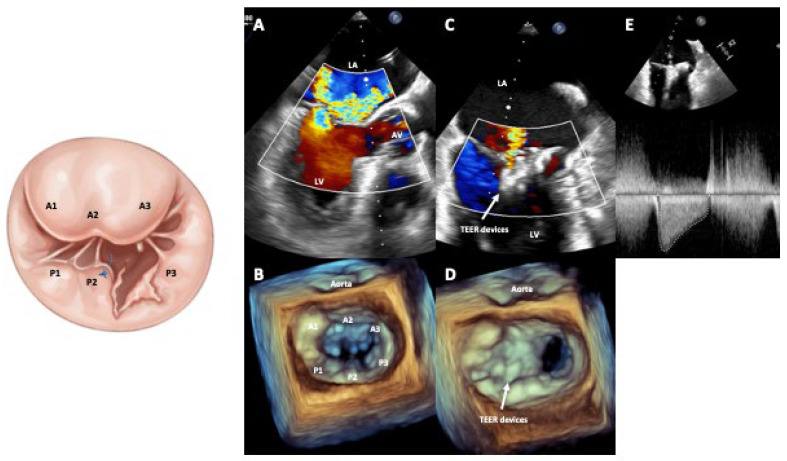
Transcatheter Edge-to-Edge Repair in Alfieri Stitch dehiscence. (**A**) TEE with Color Doppler showing severe mitral regurgitation characterized by a double jet into the left atrium (LA). The left ventricle (LV) and aortic valve (AV) are visualized. (**B**) Three-dimensional TEE atrial en-face view of the mitral valve, demonstrating residual tissue from a prior surgical Alfieri Stitch on P2 and a concomitant cleft on P2–P3. (**C**) Color Doppler imaging after transcatheter edge-to-edge repair (TEER) showing marked reduction of mitral regurgitation; the arrow indicates the implanted TEER devices. (**D**) Post-procedural 3D TEE atrial “en-face” view confirming device position. (**E**) Continuous-wave Doppler across the mitral valve after TEER demonstrating a mild increase in transmitral gradient. LA = left atrium; LV = left ventricle; AV = aortic valve; TEER = transcatheter edge-to-edge repair.

Percutaneous strategies for mitral valve repair are an alternative to avoid surgery in selected high-risk patients. Transcatheter edge-to-edge mitral valve repair (mTEER) is performed via a transseptal approach, with one or more clips used to approximate the free edges of the anterior and posterior leaflets, replicating the Alfieri edge-to-edge surgical technique [27]. A study including 104 patients treated with mTEER following a previous MVr showed device success of 89%, an in-hospital mortality of 2%, and a mean length of stay of 3 days. Although device success was slightly lower than the 91–96% reported in a series of native MR, this study demonstrated the feasibility and safety of mTEER in the setting of recurrent MR after surgery [28]. A meta-analysis by Xu et al. [8] reviewed eight single-arm studies including 212 patients with recurrent MR after failed repair: 197 underwent MitraClip implantation and 15 received a NeoChord device. The pooled Society of Thoracic Surgeons (STS) score was 6.7%, and the pooled EuroSCORE was 13.2%. At first follow-up, 83% of patients were in NYHA functional class I–II, 90% had ≤moderate MR, and 68% had ≤mild MR, with an in-hospital mortality lower than 1%. The 2024 GIOTTO FAILS study by Giordano et al. [29] analyzed 2238 patients undergoing mTEER and found that 40 (1.8%) had previous surgical mitral valve surgery (including 8 with prior annuloplasty). Device and procedural success were 95.0% and 95%, respectively. Exploratory adjusted analyses, although limited by the small sample sizes of the non-naïve groups, showed outcomes comparable to naïve patients across key endpoints, including all-cause and cardiac mortality and all-cause and heart failure rehospitalization (all *p* > 0.05). These results indicate that mTEER can be offered to patients with prior mitral interventions, with favorable expectations for functional and echocardiographic improvement. However, challenges related to impaired leaflet visualization due to echocardiographic shadowing from the annuloplasty ring and potential clip interference related to annular downsizing—both of which may predispose to mitral stenosis—should be taken into account [30]. Despite these limitations, overall procedural success rates have been high, with no significant differences between patients previously treated with ring annuloplasty and those without a ring (Figure 2). A summary of the available studies evaluating the role of mTEER after failed SMVr is shown in Table 5. Known factors identified in previous studies and recognized as favorable or unfavorable for mTEER in the setting of failed prior surgical MVr [8,10,31,32] are summarized in Table 6. It should be noted how, even in the presence of different and more complex surgical techniques (i.e., previous surgical Alfieri stitch), provided that enough leaflet and enough valve area are left, mTEER is feasible and can obtain excellent procedural results (Figure 3).

Despite recognized anatomical limitations, overall procedural success rates with mTEER in failed surgical repairs have been reported as high. However, it should be acknowledged that definitions of device success, technical success and MR reduction vary across studies, which may influence the interpretation and comparability of reported outcomes.

## 6. Percutaneous Approach: Transcatheter Mitral Valve Replacement (TMVR)

Transcatheter mitral valve replacement (TMVR) is an emerging option for failed surgical interventions, although comparative data remain limited [34]. The SAPIEN 3 valve has been widely tested in this setting in high surgical-risk patients [35]. Reported 30-day and 1-year mortality rates range from 0–8% and 11–16%, respectively, with >95% procedural success [16,17,36,37,38,39,40,41,42,43]. A key limitation is the risk of left ventricular outflow tract obstruction (LVOTO), which may be fatal and is more likely when the projected neo-LVOT area is <1.7 cm^2^ and in patients with higher ejection fraction [16,33,43,44,45]. Patient selection therefore relies on multidisciplinary Heart Team evaluation integrating surgical risk (STS/EuroSCORE, frailty, comorbidities) with detailed computed tomographic assessment of the mitral apparatus and LVOT, excluding patients with active endocarditis, prosthetic dehiscence, atrial septal thrombosis, or prohibitive LVOTO risk. Ring characteristics (rigidity, shape, and fluoroscopic visibility) should also be taken into account, as they affect anchoring, sealing, and gradients [33]. To date, the largest TMVR series, which included 141 MVIR procedures, procedural success was 80.9%, with 30-day and 1-year mortality rates of 9.9% and 30.6%, respectively [43]. Patient selection for MVIR is usually restricted to very high surgical-risk patients with suitable ring anatomy (preferably complete, rigid or semirigid, and fluoroscopically visible) and without prohibitive mechanisms of failure (e.g., extensive dehiscence, uncontrolled infection, or severe paravalvular leak) [34].

Careful multimodality imaging assessment is crucial to predict and prevent LVOT obstruction before TMVR. In addition to annular sizing and neo-LVOT estimation by cardiac computed tomography, evaluation of anterior leaflet length, septal hypertrophy, aorto-mitral angulation, and ventricular cavity dimensions is essential to identify patients at prohibitive risk of obstruction [44,45]. In selected high-risk anatomies, dedicated adjunctive techniques aimed at modifying or lacerating the anterior mitral leaflet before valve implantation may be required to maintain LVOT patency. Recent advances in transcatheter electrosurgical techniques have significantly expanded the anatomical eligibility for TMVR in patients previously considered unsuitable because of prohibitive LVOT obstruction risk or complex post-surgical anatomy. Among these, the LAMPOON (Laceration of the Anterior Mitral leaflet to Prevent Outflow ObstructioN) technique consists of intentional electrosurgical splitting of the anterior mitral leaflet immediately before TMVR in order to preserve blood flow through the LVOT after valve implantation. More recently, the BATMAN (“Balloon-Assisted Translocation of the Mitral Anterior leaflet”) technique combines leaflet traversal, balloon-assisted displacement, and electrosurgical modification to improve leaflet opening and reduce the risk of LVOT obstruction during TMVR.

Overall, the development of electrosurgical adjunctive techniques is progressively shifting the anatomical boundaries of TMVR, allowing treatment of patients previously deemed unsuitable for transcatheter intervention. However, these procedures remain technically demanding and require advanced expertise, meticulous multimodality imaging planning, and integration within experienced structural Heart Teams.

Furthermore, previous surgical or transcatheter edge-to-edge repair may represent an additional technical challenge in failed mitral repair undergoing TMVR [46]. The presence of leaflet approximation devices or surgical Alfieri stitches may alter leaflet mobility, increase the risk of device malapposition, and complicate valve deployment. In these scenarios, dedicated leaflet-modification strategies like the ELASTA-clip technique [47], which aims to liberate the anterior mitral leaflet from a surgical coaptation stitch or transcatheter clip device to create a single orifice before TMVR. The clip or stitch may be accessed antegrade via a transseptal approach or retrograde from the femoral arteries. Catheters are positioned in each mitral valve orifice, and a guidewire is passed from one and snared from the other. The Flying V is positioned along the anterior mitral valve leaflet edge of the clip or stitch, which is lacerated during 1 to 5 s of radiofrequency energy application at 70 W with a continuous dextrose infusion. The clip or stitch remains on the posterior mitral valve leaflet, and TMVR is performed with no further issues.

## 7. Discussion

Management of failed mitral valve repair has evolved substantially over the past two decades. Observational data showed that SMVr is usually favored whenever technically feasible, SMVR is reserved for anatomically unsuitable valves or advanced disease, and transcatheter therapies are considered in patients with prohibitive or high surgical risk. The choice among these options requires integration of clinical, anatomical, and procedural variables rather than reliance on a single parameter. Although observational data have historically suggested a hierarchical approach, this may oversimplify contemporary decision-making. In current practice, decisions are not linear but should always be anatomy-driven and risk-adjusted.

Timing and mechanism of failure are central to decision-making. Early failures, often driven by technical issues such as suture or ring dehiscence or SAM, are more amenable to re-repair, with outcomes approaching those of primary surgery when performed in experienced centers. Late failures, in contrast, frequently reflect disease progression or leaflet fibrosis and are more often treated with SMVR. Comorbidities, LVEF, pulmonary hypertension, and concomitant coronary or valvular disease further modulate the balance between benefit and risk for repeat surgery. Among them, the presence of concomitant tricuspid regurgitation represents an important and often underrecognized factor influencing the management of patients with failed mitral valve repair. Significant tricuspid regurgitation is frequently observed in this population, particularly in the setting of long-standing left-sided valve disease, pulmonary hypertension, and right ventricular remodeling, and has been consistently associated with worse clinical outcomes if left untreated [48]. From a pathophysiological perspective, tricuspid regurgitation may both reflect and contribute to advanced stages of cardiac remodeling, thereby identifying a subgroup of patients with more complex disease and higher procedural risk. In the context of failed MVr, the presence of significant tricuspid regurgitation may therefore favor a surgical approach, particularly when redo surgery is otherwise feasible, allowing for concomitant tricuspid valve repair and more comprehensive correction of valvular disease. Conversely, transcatheter strategies targeting only the mitral valve may leave residual right-sided pathology untreated, potentially limiting symptomatic improvement and long-term outcomes.

In our practice, the presence and severity of tricuspid regurgitation are systematically integrated into the decision-making process, with particular attention to right ventricular function, pulmonary pressures, and annular dilatation. Patients with significant tricuspid regurgitation and acceptable surgical risk are preferentially considered for redo surgery with concomitant tricuspid repair, whereas in high-risk patients, a tailored transcatheter strategy may be pursued, potentially including staged mitral and tricuspid interventions in experienced centers.

Percutaneous interventions have expanded the therapeutic armamentarium for patients with failed MVr, particularly in older or comorbid populations. mTEER has demonstrated high procedural success, low early mortality, and durable MR reduction in selected patients with prior surgery, although device success remains slightly lower than in native valves and the risk of mitral stenosis is non-negligible, especially in the presence of complete, downsized rings. In the presence of more complex surgical anatomies, a higher expertise is usually required to undergo effective mTEER. Since imaging guidance is usually the most critical issue to be solved, careful imaging planning, possibly including adjunctive techniques (i.e., ICE, pre-procedural CT), may be required to understand the feasibility of the procedure and to guide the strategy.

Overall, current evidence is derived largely from retrospective series, registries, and heterogeneous cohorts. Direct comparisons between re-repair, SMVR, mTEER, and TMVR are limited and subject to substantial selection and referral bias. Nonetheless, the available data support a tailored strategy to be discussed in multidisciplinary Heart Teams after having collected high-quality and often multiple source images to better define valve anatomy and possibly plan the most suitable therapeutic option. At our high-volume valve center, the management of failed mitral valve repair has progressively shifted from a conventional stepwise paradigm to a fully integrated, anatomy-driven strategy in which surgical and transcatheter options are considered upfront and on equal footing within a dedicated Heart Team framework.

In this setting, multimodality imaging—systematically incorporating high-resolution transesophageal echocardiography and cardiac computed tomography—is pivotal not only for diagnosing the mechanism of failure but also for anticipating procedural feasibility and guiding treatment allocation. This approach allows early identification of patients who are unlikely to benefit from redo surgery despite acceptable operative risk, as well as those in whom transcatheter strategies may provide a more predictable and less invasive solution. As a result, decision-making is deliberately non-linear and strongly anatomy-oriented: early technical failures with preserved leaflet integrity are preferentially directed toward surgical re-repair, whereas complex or mixed mechanisms, particularly in the presence of leaflet restriction, prior extensive resection, or annular distortion, frequently prompt consideration of transcatheter approaches even beyond traditional high-risk categories.

Within this evolving paradigm, mTEER has emerged not merely as an alternative for inoperable patients but as a cornerstone therapy in selected cases of failed repair. In our experience, its role is particularly relevant in patients with focal regurgitant jets, sufficient residual leaflet tissue, and favorable valve area, where it can achieve consistent MR reduction with minimal physiological impact. Importantly, increasing operator experience and procedural standardization have significantly improved feasibility even in challenging post-surgical anatomies, including prior annuloplasty rings and edge-to-edge repairs. Looking ahead, the role of mTEER is expected to expand further in the immediate future, driven by ongoing refinements in device technology, enhanced imaging integration, and a growing body of real-world evidence. In this context, mTEER may progressively move from a rescue or second-line therapy to an early, anatomy-driven option in carefully selected patients with failed MVr, particularly when surgical durability is uncertain or when repeat surgery carries a disproportionate burden in terms of invasiveness and recovery. Nevertheless, this expansion should not be interpreted as a shift toward indiscriminate use. As a fact, TEER failure still represents an issue that is mainly fixed by mitral valve replacement [49]. Although transcatheter therapies have substantially expanded the treatment options for patients with failed mitral valve repair, the favorable procedural and short-term outcomes reported with mTEER should not be interpreted as evidence of equivalence with surgery. In contemporary practice, mTEER is primarily offered to patients who are older, frailer, and at higher surgical risk, which inherently complicates direct comparisons between treatment strategies. Nevertheless, when a durable surgical repair or replacement can be safely performed, surgery remains the reference treatment and is generally associated with superior correction of mitral regurgitation, lower rates of residual or recurrent MR, and greater long-term durability [50,51].

## 8. Future Perspectives

Future advances in the management of failed mitral valve repair are likely to arise from several complementary directions. First, improvements in multimodality imaging—including high-resolution three-dimensional transesophageal echocardiography, cardiac computed tomography, and intra-cardiac echocardiography—will further refine preprocedural planning for both surgical and transcatheter interventions. Second, prospective registries and, where feasible, randomized or carefully controlled comparative studies will be needed to better define the relative benefits of SMVr, SMVR, mTEER, and TMVR across different patterns of failure and risk profiles. Standardized definitions of repair failure, procedural success, and clinically meaningful endpoints will be essential to allow robust comparisons across studies. Finally, computational modeling, artificial intelligence–based risk prediction, and patient-specific simulations may help Heart Teams anticipate procedural outcomes, optimize device selection, and personalize therapy. In particular, AI-based learning algorithms can integrate clinical, echocardiographic, computed tomography, and procedural variables to improve risk stratification, predict repair durability, and support treatment allocation by facilitating identification of the underlying mechanism of failure, assessment of anatomical suitability for transcatheter therapies, and prediction of complications. Furthermore, AI-based computational modeling and digital twin technologies may enable patient-specific simulation of different therapeutic strategies before intervention. Although current evidence remains preliminary and prospective validation is still required, AI has the potential to enhance Heart Team decision-making and further support a personalized approach.

## 9. Limitations

A number of limitations should be acknowledged. First, the available evidence on the management of failed mitral valve repair is largely derived from observational studies, registries, and single-center experiences, with a lack of randomized controlled trials directly comparing surgical re-repair, surgical replacement, and transcatheter interventions. As a result, the reported outcomes are inherently affected by selection bias, referral bias, and heterogeneity in patient populations.

Second, definitions of key endpoints—such as repair failure, procedural success, and degree of mitral regurgitation reduction—vary across studies, limiting the comparability of results and the ability to perform robust quantitative synthesis.

Third, treatment allocation in most studies reflects real-world clinical decision-making and is strongly influenced by patient risk profile, anatomical suitability, and institutional expertise, which may further confound comparisons between treatment strategies.

## 10. Conclusions

Failed mitral valve repair is increasingly encountered in contemporary practice and is associated with substantial morbidity and mortality; when technically feasible, surgical re-repair remains the preferred strategy, particularly in early failures driven by correctable technical causes and in younger, lower-risk patients, whereas SMVR is indicated in the presence of mitral stenosis, extensive leaflet pathology, endocarditis, multivalve disease, or anatomy unsuitable for re-repair. In patients at high or prohibitive surgical risk, transcatheter therapies—including mTEER for recurrent MR with carefully evaluated suitable anatomy—should be considered within a multidisciplinary Heart Team framework that emphasizes patient-centered, anatomy-driven decision-making. The treatment strategy should not follow a rigid hierarchy but rather an individualized, anatomy-driven and risk-adjusted approach defined by the Heart Team.

## Figures and Tables

**Table 1:** Causes of Mitral Valve Repair Failure classified according to the prevalent mechanism.

Specific Cause	Description	Timing
*Technical failure*, 43% of cases [10]		
Suture/Annuloplasty Dehiscence	Dehiscence of annuloplasty sutures/at the site of leaflet resection or disconnection of artificial chords. May result in hemolysis due to the regurgitant jet impacting the loose annuloplasty.	Early postoperative period
Chordal Pseudo-elongation	Artificial chordae become too long after LV remodeling/ shrinking post-repair. To prevent this, chords to the posterior leaflet should be made relatively short during the initial repair	Early/mid postoperative period
Systolic Anterior Motion (SAM)	Anterior movement of the mitral valve towards the IVS during systole, causing dynamic LVOTO and secondary MR. Occurs when the zone of coaptation is too close to the septum.	Early postoperative period/after ventricular remodeling
Incomplete Repair [13]	Residual prolapse with >1+ MR, leaflet restriction, SAM, or mitral stenosis due to restrictive annuloplasty/extensive leaflet suturing.	Immediate postoperative period
*Valve-related failure*, 56% of cases [10]		
Disease Progression	Development of new prolapse in previously healthy leaflet areas due to chordal elongation or rupture. Most common cause of recurrent MR in both early and late failures.	Late postoperative period
Progressive Fibrosis/Scarring	Formation of scarring related to suture lines, leading to stiff, non-pliable leaflets and functional mitral stenosis, after excessive resection/plication of the posterior leaflet	Late postoperative period
Progressive LV Dilatation	LV enlargement causing restriction of leaflet motion and development of recurrent prolapse (the chordae become too long for the remodeled ventricle) or secondary MR	Late postoperative period
Endocarditis	Bacterial/fungal infection of the mitral valve resulting in tissue destruction and annular invasion. Historically managed with replacement, but repair options are evolving.	Variable

IVS = interventricular septum; LV = left ventricle; LVOTO = left ventricle outflow tract obstruction; MR = mitral regurgitation; SAM = systolic anterior motion.

**Table 2:** Clinical and echocardiographic predictors of feasibility of mitral valve re-repair.

**Unfavorable Factor for Re-Repair**	**Favorable Factors for Re-Repair**
Older age Chronic kidney disease Coronary artery disease Echocardiographic Parameters: coaptation depth > 1 cm systolic tenting area > 2.5 cm^2^ posterior mitral leaflet angle > 45° distal anterior mitral leaflet angle > 25° end-systolic interpapillar distance > 2 cm systolic sphericity index > 0.7 PAPs ≥ 50 mmHg	Shorter time to reoperation (strongest association to efficacy of re-repair) Younger age Operative findings in early failure suture dehiscence, adverse ventricular remodeling

PAPs = pulmonary artery systolic pressure.

**Table 3:** Mechanism-based management strategies in failed mitral valve repair (according to etiology).

Mechanism of Failure	Re-Repair	Replacement
Failed posterior neochordae (D)	+ (resection)	
New areas of prolapse for rupture/elongation (D)	+ (neo-chordae, avoid resection)	
Adverse ventricular remodeling (F)	+ (chord shortening)	
Suture dehiscence (DF)	+	
New area of prolapse in the commissure (D)	+ (closure of the commissure)	
Valvular disease progression (D)		+
Endocarditis (DF)		+
Mitral stenosis (DF)		+

D = degenerative; F = functional.

**Table 4:** Mitral valve repair options.

Cut & Sew	Respect Rather than Resect
Quadrangular/triangular resection Posterior leaflet sliding Chordal replacement/flip over	PTFE chordae/neochord Edge-to-edge (Alfieri stitch) Chordal shortening Papillary muscle replacement Annuloplasty

PTFE = polytetrafluoroethylene.

**Table 5:** MitraClip for failed SMVr in various studies.

	Grasso 2014	Estevez 2016	Braun 2017	Niikura 2019	Rahhab 2021	H. Xu 2022	Giordano 2024
Cause of pre-operative MR							
Degenerative		33%	39%		50%		67.5%
Functional		67%	52%	92%	35%		27.5%
Mixed			9%	8%	8%		5%
Repair technique							
Annuloplasty	100%	100%	79%		90%		8%
Chordal repair					13%		
Edge-to-edge			9%		16%		
Resection					8%		
Combined			21%		28%		
Cause of recurrent MR							
Ring dehiscence/rupture			23%	8%	7%		
Flail/leaflet prolapse			50%	83%			
SAM				8%	3%		
Functional			27%		39%		
Procedural outcomes							
Device success *	100%		84%	67%	89%		95%
Technical success #			84%		90%		95%
Significant MR reduction §	50%		82%	83%	94%	96%	90%
In-hospital mortality			0		2%	<1%	0
6-months mortality			6%		6%		
1-year mortality			18%		9%		12.5%
NYHA I-II at first follow-up			66%	75%	85%	83%	

* Device success = procedural efficacy = proper placement of the device without procedural mortality + reduction in postprocedural MR by ≥1 grade from baseline and to an absolute level of moderate-higher MR; # Technical success = procedural safety = successful deployment of the device + absence of procedural mortality and freedom from emergency surgery; § Significant MR reduction = reduction in postprocedural MR by ≥1 grade from baseline. MR = mitral regurgitation; NYHA = New York Heart Association; SAM = systolic anterior motion.

**Table 6:** Favorable and unfavorable factors for mTEER in failed SMVr.

Favorable Factors	Unfavorable Factors
Alfieri stitch detachment without leaflet perforation	Endocarditis (contraindication) Leaflet perforation/tear
Posterior leaflet length	Poor acoustic window
Factors contraindicating MVIV/MVIR Large septal bulge Small aorto-mitral angle < 120 degrees;	Ring dehiscence: ring can obstruct repair, as it can interfere with the clip delivery system trajectory
Central jet origin (A2–P2 zone)	Wide residual pathology
EVEREST Criteria [33] Coaptation length > 2 mm Coaptation depth < 11 mm Flail gap < 10 mm Flail width < 15 mm MVA > 4 cm^2^ Mobile leaflet length > 1 cm	Severe calcification in the grasping area Very short residual leaflet (<6 mm) with or without ring dehiscence Severe leaflet tethering
Annular dilatation	Baseline diastolic gradient > 5 mmHg

mTEER = mitral transcatheter edge-to-edge repair; MVA = mitral valve area; MVIR = mitral valve-in-ring; MVIV = mitral valve-in-valve; SMVr = surgical mitral valve repair.

## Data Availability

No new data were created or analyzed in this study.

## Abbreviations

IVS	Interventricular septum
LV	Left Ventricle
LVOTO	Left ventricular outflow tract obstruction
MR	Mitral regurgitation
mTEER	Transcatheter mitral edge-to-edge repair
MVIR	Mitral valve-in-ring
MVIV	Mitral valve-in-valve
MVR	Mitral valve replacement
MVr	Mitral valve repair
SMR	Secondary (functional) mitral regurgitation
SMVR	Surgical mitral valve replacement (after failed repair)
SMVr	Surgical mitral valve re-repair
TEER	Transcatheter edge-to-edge repair
TMVR	Transcatheter mitral valve replacement
